# Stroke survivors’ preferences on assessing patient-reported outcome measures

**DOI:** 10.1186/s41687-023-00660-1

**Published:** 2023-11-30

**Authors:** Richard Schmidt, Daniela Geisler, Daniela Urban, Rebecca Pries, Christina Franzisket, Christian Voigt, Galina Ivanova, Thomas Neumuth, Joseph Classen, Markus Wagner, Dominik Michalski

**Affiliations:** 1 Department of Neurology, University of Leipzig, Liebigstr. 20, 04103 Leipzig, Germany; 2 German Stroke Foundation, 33330 Gütersloh, Germany; 3 Innovation Center Computer Assisted Surgery (ICCAS), University of Leipzig, 04109 Leipzig, Germany

**Keywords:** Patient-reported outcome measures, PROMs, Cerebral ischemia, Stroke aftercare

## Abstract

**Background:**

To assess quality of life and unmet needs after stroke, patient-reported outcome measures (PROMs) have gained increasing attention. However, patients’ perspectives on assessing PROMs remain unclear, potentially hindering implementation into clinical practice. Therefore, this study explored patients’ preferences on assessing PROMs after ischemic stroke.

**Methods:**

A paper-based questionnaire was sent to stroke survivors treated at the Department of Neurology, University of Leipzig, Germany. Health-related quality of life (HRQoL, EQ-5D-5L) and preferences regarding different aspects of data collection to assess PROMs were investigated and linked to socio-demographic and medical characteristics.

**Results:**

158 persons were contacted and 80 replies were subsequently analyzed. Mean age was 70.16 years and mean HRQoL was 68.79 (visual analogue scale with a theoretical maximum of 100). Participants showed positive attitudes towards PROMs as they saw potential to improve care of other patients (n = 66/79; 83.54%) or to improve their own situation (n = 53/74; 71.62%). Participants preferred an annual interview after stroke (n = 39/80; 48.75%) and would preferably spend 15–30 min (n = 41/79; 51.90%) to answer a written survey (n = 69/80; 86.25%). The initially treating clinic was preferred as initiator of such surveys (n = 43/79; 54.43%). Stratification revealed that participants with more than 1 h of daily digital media usage preferred email as way of communication.

**Conclusions:**

For the first time, this study showed individual preferences on assessing PROMs after ischemic stroke, focusing on the way, time interval, duration, and initiation site of surveys. These insights might help to successfully implement PROMs after stroke and subsequently detect unmet needs and deficits in stroke care.

**Supplementary Information:**

The online version contains supplementary material available at 10.1186/s41687-023-00660-1.

## Introduction

While more than 101 million people worldwide are currently affected by stroke, approximately 12 million people suffer a stroke each year [1]. Due to significant progress in stroke prevention, acute therapy, and rehabilitation, the relative proportion of fatal strokes appears to have decreased in recent years [2].

However, with ongoing demographic change and reduced stroke-related mortality, a growing number of people are living with long-term consequences of stroke [1, 3]. In detail, physical functioning, the health-related quality of life (HRQoL), and general well-being of stroke survivors may be significantly reduced. Moreover, in affected individuals, physical, cognitive, and emotional impairments can negatively impact the ability to participate in activities of daily living or return to work [4–7]. For this reason among others, the Stroke Action Plan for Europe was recently introduced by the European Stroke Organization (ESO) and the Stroke Alliance for Europe as patient organization [8]. Consequently, research has begun to explore patients’ individual experiences after stroke, including, for instance, HRQoL [9]. In an attempt to display the results of treatments and the impact of stroke on HRQoL from the perspective of stroke survivors, patient-reported outcome measures (PROMs) have increasingly gained attention in stroke care and related research [10]. Here, PROMs can provide valuable insights into the individually experienced impairment in physical, social, and psychological dimensions following stroke and thus help to optimize acute stroke treatment and aftercare [11, 12]. In addition, PROMs could improve individualized treatment planning during the follow-up process by using their results for immediate therapy adjustment.

Despite this increasingly recognized importance of PROMs in stroke research, their use is still limited and has not yet been implemented into clinical practice [13]. One reason might be that the most effective way to assess PROMs on a regular basis has yet to be investigated. For example, scarce evidence exists on the patient perspective when or how to conduct related surveys after an ischemic stroke in order to receive reliable and more generally valid results [14]. Moreover, consensus about the choice of PROMs that might be most appropriate for use in a population affected by ischemic stroke is missing [15]. Consequently, the heterogeneity in using PROMs currently prevents conclusions about which media are most appropriate for distribution of PROMs and at which time points interviews should take place. As exemplarily illustrated by a study in patients with chronic kidney disease, the optimal way for assessing PROMs seems to depend on individual factors, as, for instance, electronic PROM surveys (ePROMs) were applicable only for certain subpopulations [16]. However, comparable investigations are missing in the field of stroke, leading to a strong need for further information on using PROMs in stroke patients [8, 17]. Therefore, it seems necessary to involve the public beyond a professional community and, in particular, to include affected person themselves [18, 19]. The involvement of affected individuals in stroke research can be beneficial for themselves, their families, and the quality of scientific investigations and is therefore considered advantageous to the research process [20].

This study thus explored patients’ preferences regarding PROMs after ischemic stroke, mainly by investigating how, when, in what form, and by whom they should be initiated and implemented. Preferences were further examined in the context of clinical and psychosocial parameters to consider possible associations between the opinions and characteristics of those affected by ischemic stroke. The emerging information could help to plan future initiatives towards a systematic use of PROMs after stroke and could thus contribute to an improved stroke care.

## Materials and methods

### Study design

This exploratory study recruited participants of a local stroke pilot program, who had been hospitalized for treatment of ischemic stroke or transient ischemic attack (ICD codes I63.* and G45.*) at the stroke unit of the Department of Neurology at the University of Leipzig between January 2020 and January 2022. Each patient eligible for recruitment received a letter describing the background of the study and a paper-based, five-page questionnaire with 30 questions. First letters were sent out on March 1, 2023, and replies received until May 5, 2023, were included in the analyses. Upon receipt, replies were digitalized and linked to clinical patient data from time of stroke unit treatment.

This study was conducted in accordance with the guidelines of the Declaration of Helsinki and was approved by the institutional review board of the Medical Faculty of Leipzig University (reference number 019/23-ek). Written informed consent was obtained from all participants. The study was registered in the German Clinical Trial Register (reference number DRKS00031333). Reporting of results considered the STROBE statement guidelines for cross-sectional studies [21].

### Data

National Institutes of Health Stroke Scale [22] (NIHSS) at hospital admission and discharge and modified Rankin Scale [23] (mRS) at discharge were retrospectively collected from patients’ records from time of stroke unit treatment. In addition, socio-demographic data such as current relationship status, employment status, and education were taken from patients’ records. The paper-based questionnaire included a single-page version of the EQ-5D-5L to assess HRQoL, while index values were calculated using the German standard value set [24, 25]. Use of EQ-5D-5L with a minor adaption [visual analogue scale (VAS) was displayed horizontally instead of vertically to save space] was confirmed and permitted by EuroQol. Outcome parameters in terms of preferences on assessing PROMs were captured by a specially designed four-page questionnaire (translated version in Additional file 1).

### Statistical analyses

Socio-demographic and clinical baseline characteristics were calculated using descriptive statistics. For subgroup analyses, answers on preferred circumstances of assessing PROMs were stratified for HRQoL (using mean of EQ-5D-5L index value), the degree of stroke-related symptoms at time of hospital discharge (using NIHSS at time of hospital discharge), mean age at time of stroke, and daily usage of digital media (assessed in hours per day). Mean was used for stratification because multiple responses would have been precisely median, and in these cases stratification to one group would not have been possible. Respective proportions were compared for statistically significant differences with Chi-Square test for equality of proportions and continuity correction if necessary. Significance levels for all the statistical tests were set to *p* < 0.05. Data processing and all analyses were performed using R Statistical Software with R Studio [26, 27].

## Results

### Patients’ characteristics

Of 158 patients who had been contacted by letter, 83 (52.53%) completed the questionnaires within the study period. Of all replies, questionnaires from patients with transient ischemic attack (n = 3; 3.61%) were excluded from analyses as this subgroup was too small for comparison in subgroup analyses. Therefore, this study was based on 80 patients who had experienced an ischemic stroke. Baseline characteristics of these patients are given in Table 1. Participants’ mean age was 70.16 years and at time of completing the questionnaire a mean of 112.83 weeks had passed since the qualifying ischemic event. Regarding psychosocial characteristics, most patients were retired and lived in a relationship, while HRQoL was rated relatively good as indicated by a mean EQ-5D-5L VAS of 68.79 (range 0–100). Among the dimensions affecting individual HRQoL, “pain or discomfort” (mean 2.05) was mentioned as the most present issue, followed by an impairment of “mobility” (mean 1.84) and “usual activities” (mean 1.83).

**Table 1:** Patients’ characteristics

Variable			Unit
Age (in years)	70.16	11.80	M, SD
Sex (female)	23	28.75	n, %
NIHSS at admission			
Total	3.61	3.69	M, SD
0	13	16.25	n, %
1–3	38	47.50	n, %
4–6	13	16.25	n, %
≥ 7	16	20.00	n, %
NIHSS at discharge			
Total	1.67	2.37	M, SD
0	28	35.44	n, %
1–3	42	53.16	n, %
4–6	7	8.86	n, %
≥ 7	2	2.53	n, %
mRS at discharge			
Total	1.79	1.03	M, SD
0	9	11.39	n, %
1	17	21.25	n, %
2	37	46.25	n, %
≥ 3	14	17.50	n, %
Weeks since stroke	112.83	23.67	M, SD
Single-person household	21	27.63	n, %
Working status			
Employed	11	14.10	n, %
Unemployed	3	3.85	n, %
Incapacitated	4	5.13	n, %
Retired	59	75.64	n, %
Relationship status (in a relationship)	65	81.25	n, %
Rehabilitation			
Outpatient	27	33.75	n, %
Inpatient	41	51.25	n, %
None	15	18.75	n, %
Digital media usage			
None	14	17.95	n, %
< 1 h / day	28	35.90	n, %
≥ 1 h / day	36	46.15	n, %
EQ-5D-5L			
Mobility	1.84	1.02	M, SD
Self-care	1.38	0.76	M, SD
Usual activities	1.83	1.11	M, SD
Pain or discomfort	2.05	0.92	M, SD
Anxiety or depression	1.60	0.81	M, SD
VAS	68.79	18.98	M, SD

*M* mean, *SD* standard deviation, *n* number, *NIHSS* National Institutes of Health Stroke Scale, *mRS* modified Rankin Scale, *VAS* visual analogue scale

### Patients’ preferences on assessing PROMs

The majority of patients showed overall favorable attitudes towards surveys assessing HRQoL as part of PROMs. Many participants thought that such surveys might improve care of those who could possibly be affected by stroke in the future (n = 66/79; 83.54%). Other participants suggested that their participation could result in a direct improvement for their own personal care (n = 53/74; 71.62%). According to answers given, patients thought that the completion of surveys assessing HRQoL could be hindered by physical health of participants (n = 39/80; 48.75%). It was also suggested that some patients might simply not be interested or motivated in participating in such surveys (n = 23/80; 28.75%).

Regarding the format and time aspects of PROM assessment, less participants preferred an open question type (n = 18/79; 23.08%) over multiple choice questions (n = 55/79; 70.51%) when being interviewed for their HRQoL. Participants favored surveys to be repeated in regular intervals (n = 55/76; 72.37%), preferably annually after the initial stroke (n = 39/80; 48.75%). With respect to the duration of such surveys, most patients would spend 15 to 30 min of time (n = 41/79; 51.90%), while a smaller proportion would spend less than 15 min (n = 17/79; 21.52%) or more than 30 min (n = 13/79; 16.46%). Remarkably, most patients would participate without receiving any financial or other compensation (n = 68/76; 86.08%).

Asked for the preferred way of assessing PROMs, 69 of 80 participants (86.25%) answered that HRQoL should be captured with a written survey, with letters being preferred (n = 58/80; 72.50%) over email (n = 17/80; 21.25%), and other ways of communication. Fifty-six of the 80 participants (70.00%) also deemed verbal surveys feasible. Thereby, the initially treating hospital was the preferred location for an interview (n = 28/80; 35.00%) over a phone call at home (n = 20/80; 25.00%), or an in-person survey at their general physician’s (GP) practice (n = 18/80; 22.50%).

Regarding the person or institution initiating PROMs, patients preferred them to be conducted by hospital staff (n = 43/79; 54.43%), by their GP (n = 32/79; 40.51%), supporting professionals in stroke aftercare, e.g., stroke pilots (n = 28/79; 35.44%), or a specialist physician (n = 22/79; 27.85%), rather than others such as scientific institutions or non-profit organizations. Most participants answered, they would also wish other people close to them to be interviewed regarding their opinion on the patient’s HRQoL (n = 47/79; 59.49%). Thereby, relatives and caregivers (n = 41/79; 51.90%) were prioritized above the GP (n = 15/79; 18.99%). Remarkably, only a minority of participants saw a need for relatives or caregivers to be interviewed about their respective HRQoL (n = 26/74; 35.14%).

About information that emerge from studies on PROMs, most participants answered, they would want to be informed about the results of a HRQoL survey they participated in (n = 57/77; 74.03%). Further, a remarkable proportion of patients would even want to be involved when future research is planned, for example, by co-developing questions (n = 30/77; 38.96%). (Overview on responses in Additional file 1)

### Patients’ preferences stratified for clinical and psychosocial factors

**Fig. 1:**
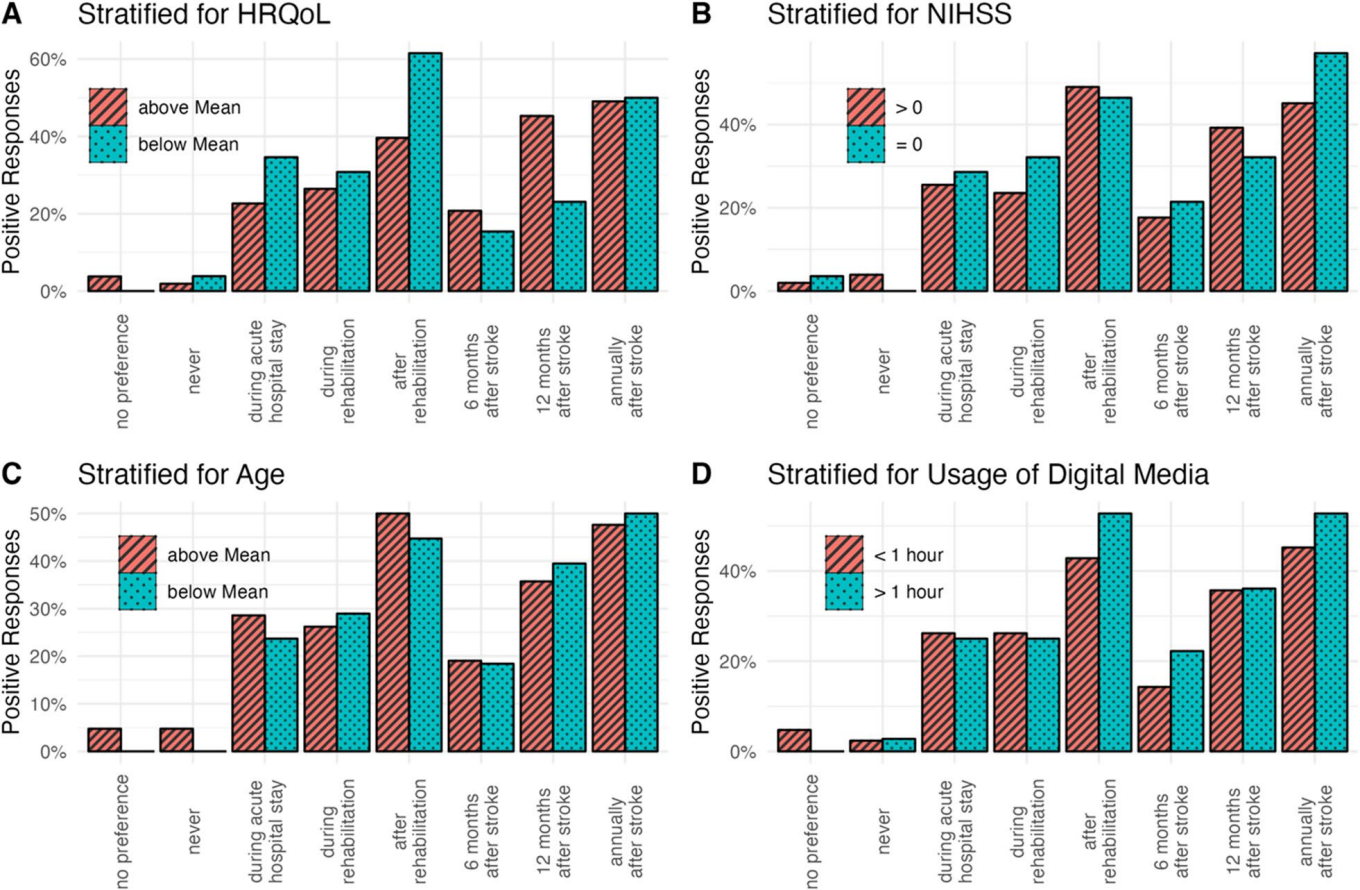
Preferred time points of PROMs surveys. Bars indicate proportion among all participants in each stratification group that chose the respective answer. Preferences were stratified for quality of life (**A**), stroke symptoms at discharge (**B**), age at stroke (**C**) and daily usage of digital media (**D**). Abbreviations: NIHSS, National Health Institutes Stroke Scale; HRQoL, health-related quality of life

**Fig. 2:**
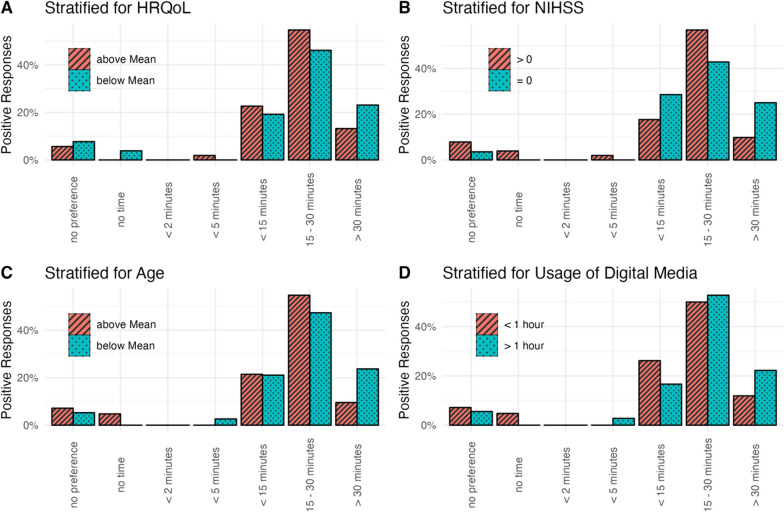
Preferred duration of PROMs surveys. Bars indicate proportion among all participants in each stratification group that chose the respective answer. Preferences were stratified for quality of life (**A**), stroke symptoms at discharge (**B**), age at stroke (**C**) and daily usage of digital media (**D**). Abbreviations: NIHSS, National Health Institutes Stroke Scale; HRQoL, health-related quality of life

No significant differences between stratification groups were found regarding the preferred time point or interval (Fig. 1) and the duration (Fig. 2) for assessing PROMs. However, while most patients preferred a duration of 15–30 min for PROMs, a trend was visible that patients with lower HRQoL, a lack of stroke symptoms, younger age, or extended usage of digital media would accept a survey of more than 30 min when compared to participants not fulfilling these criteria (Fig. 2).

**Fig. 3:**
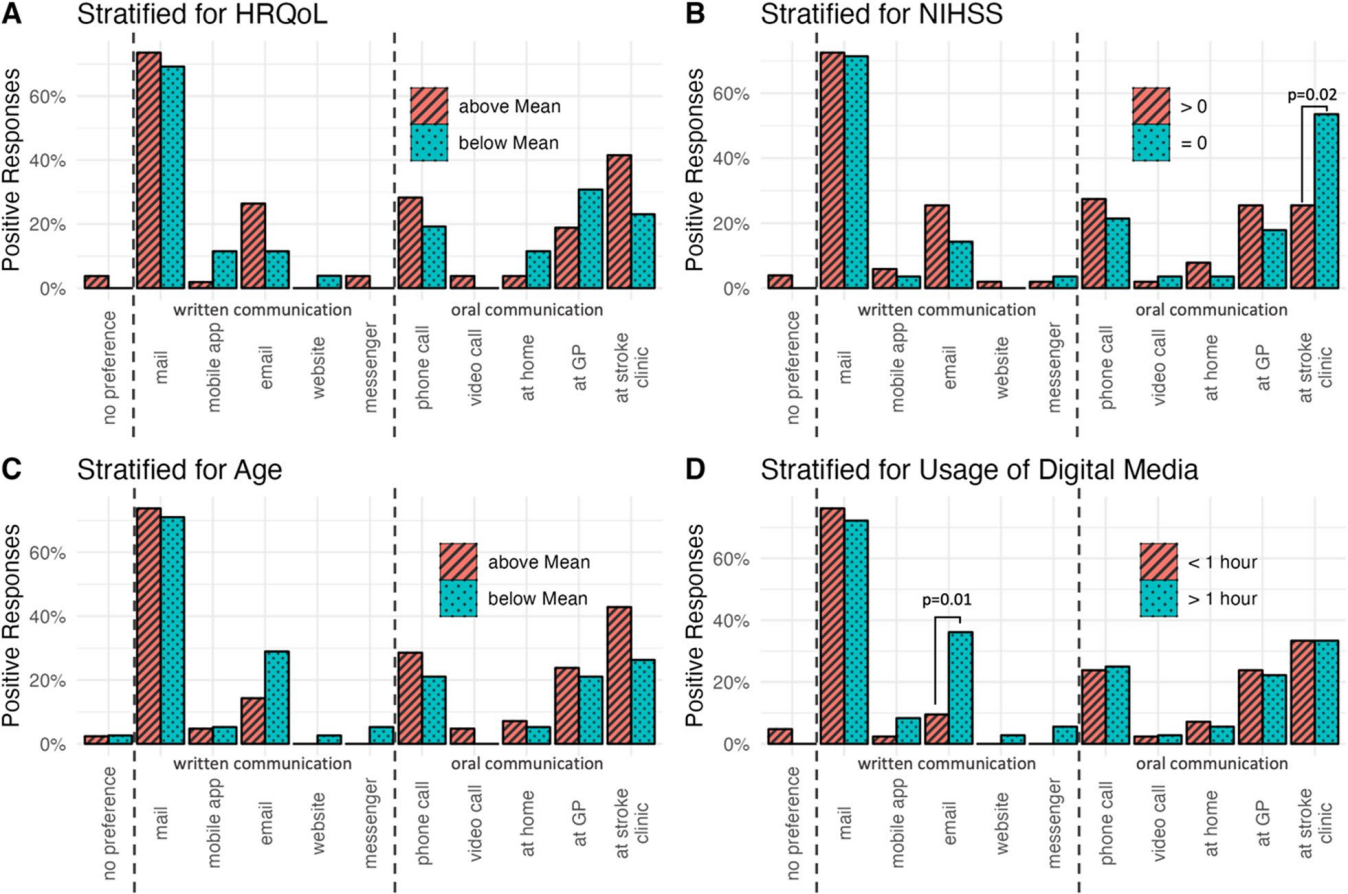
Preferred ways of communication for assessing PROMs. Bars indicate proportion among all participants in each stratification group that chose the respective answer. Preferences were stratified for quality of life (**A**), stroke symptoms at discharge (**B**), age at stroke (**C**) and daily usage of digital media (**D**). *P* values are depicted if ≤ 0.05. Abbreviations: NIHSS, National Health Institutes Stroke Scale; HRQoL, health-related quality of life; GP, general physician

**Fig. 4:**
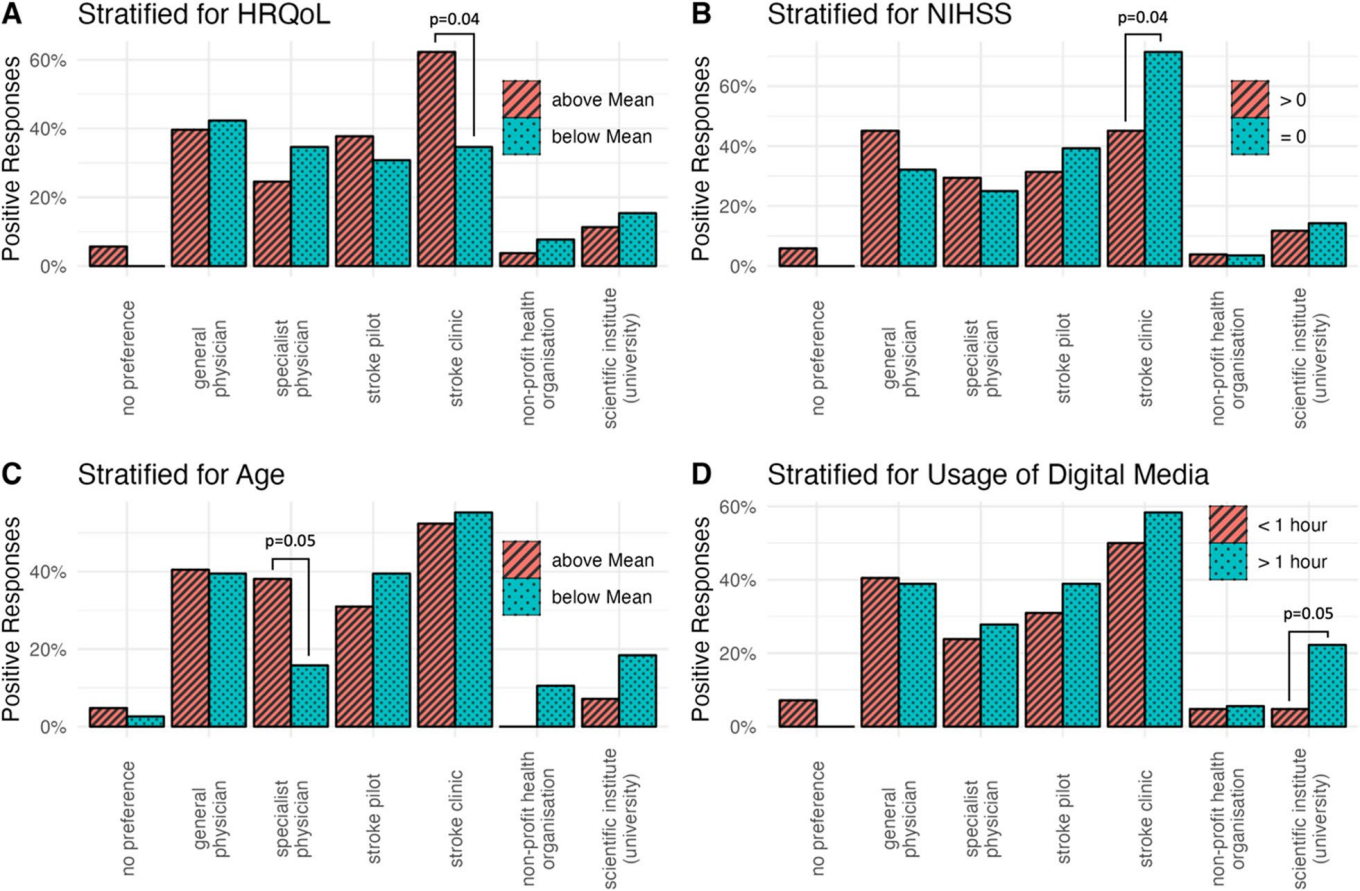
Preferred initiators for the assessment of PROMs. Bars indicate proportion among all participants in each stratification group that chose the respective answer. Preferences were stratified for quality of life (**A**), stroke symptoms at discharge (**B**), age at stroke (**C**) and daily usage of digital media (**D**). *P* values are depicted if ≤ 0.05. Abbreviations: NIHSS, National Health Institutes Stroke Scale; HRQoL, health-related quality of life

Regarding the preferred way of communication for assessing PROMs, email was chosen significantly more often by those using digital media for more than 1 h per day. Participants who were discharged with an NIHSS of 0 preferred a personal interview at the stroke clinic they had been treated at (Fig. 3). As initiators of surveys dealing with PROMs, patients without stroke symptoms (NIHSS 0) at time of hospital discharge preferred the initially treating stroke clinic significantly more often than those, who were discharged with an NIHSS of more than 0 (Fig. 4). Further, older participants (i.e., those with an age above the cohort’s mean) clearly tended to an interview with a specialist physician (e.g., neurologist) and those with a better HRQoL (i.e., reaching an index value above mean) preferred an interview by the initially treating stroke clinic significantly more often, when compared to younger participants or those with a lower HRQoL, respectively. Stratification groups did not differ significantly concerning the overall low rate of patients, who preferred an assessment on PROMs by non-profit organization. Remarkably, younger patients (i.e., those with an age below the mean) and those using digital media for more than 1 h per day, exhibited a considerable trend towards a preference for assessing PROMs by scientific institutes, when compared to older patients and those with a lesser usage of digital media.

**Fig. 5:**
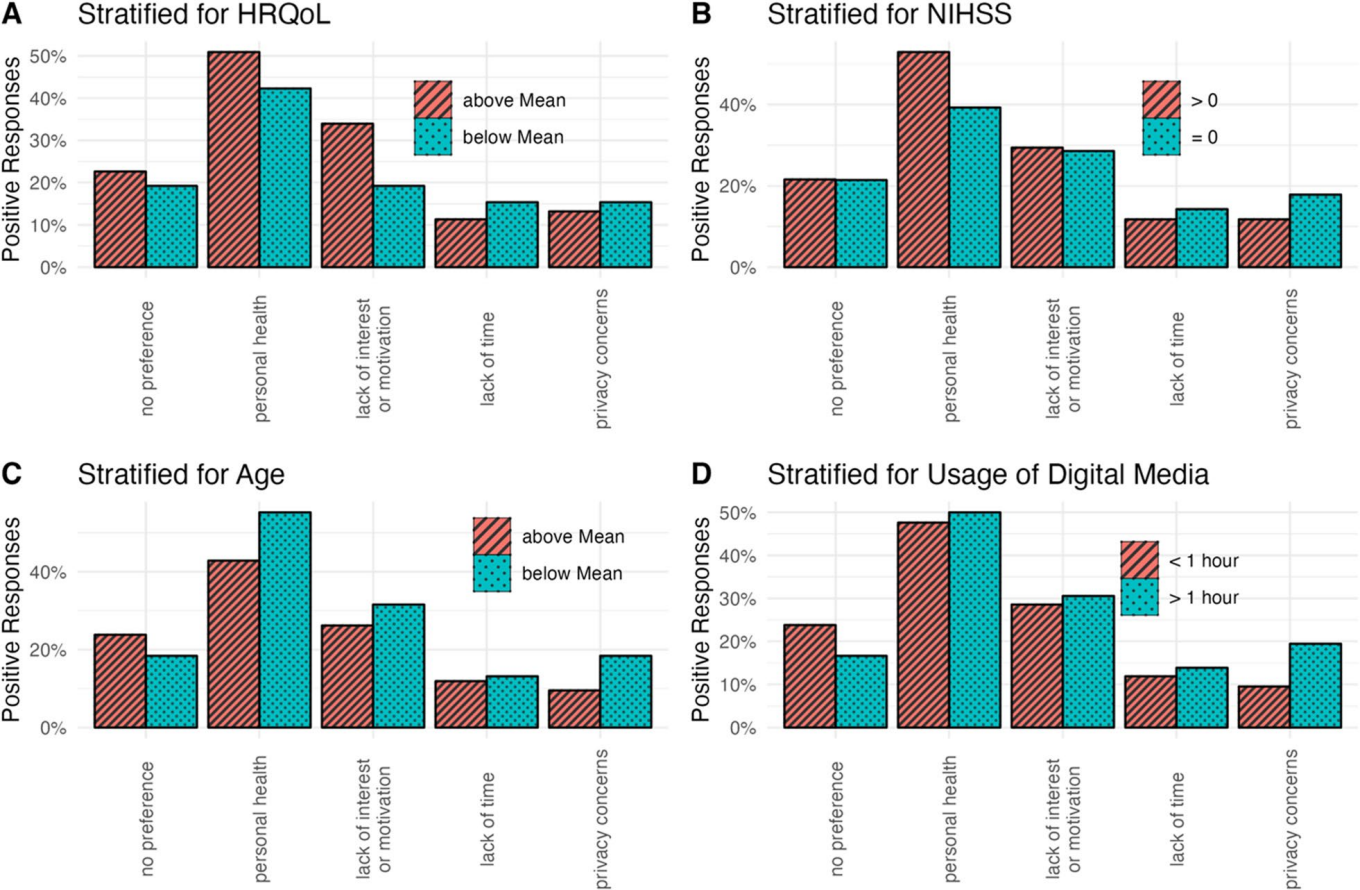
Possible barriers for assessing PROMs. Bars indicate proportion among all participants in each stratification group that chose the respective answer. Preferences were stratified for quality of life (**A**), stroke symptoms at discharge (**B**), age at stroke (**C**) and daily usage of digital media (**D**). Abbreviations: NIHSS, National Health Institutes Stroke Scale; HRQoL, health-related quality of life

Asked for barriers potentially hindering future patients to participate in PROM-related surveys, participants with better HRQoL suggested a lack of motivation could be relevant, whereas younger patients and those who use digital media more frequently named privacy concerns as a potential issue (Fig. 5). Participants with remaining stroke symptoms (NIHSS > 0 at time of hospital discharge) saw personal health as a potential barrier significantly more often than those without stroke symptoms (NIHSS 0).

## Discussion

This study aimed to explore patients’ preferences regarding the assessment of PROMs after stroke, which are increasingly seen as essential information in stroke care [8]. To strictly consider patients’ perspectives, this study applied a comprehensive questionnaire in a cohort of persons who had experienced an ischemic stroke. Furthermore, the obtained information was evaluated in the context of individual clinical and psychosocial characteristics to consider potential influencing factors. Considering that patients’ perspectives on assessing PROMs could help to implement respective surveys more successfully, the results of the present study might be valuable for future initiatives of PROMs in ischemic stroke.

As one of the main findings, most participants reported a generally favorable attitude towards PROMs after stroke. According to the resulting preferences, future investigations of PROMs should be conducted mainly by letter, use closed, i.e., multiple-choice questions, and last preferably less than 15 min but not exceed 30 min to be answered by patients. From patients’ perspective, questionnaires should be repeated over time, preferably with annual intervals. Regarding these issues, no differences were observed between stratified groups, which means that these observations might be applicable to the majority of people affected by ischemic stroke. The favorable attitude of participants towards being personally interviewed at the initially treating stroke clinic, which was particularly seen in those showing no stroke-related symptoms at hospital discharge, might be interpreted as a good trust in specialized institutions. On the other hand, this could also indicate that from the patients’ perspective, specialists should be involved in stroke aftercare. While different institutions in healthcare were seen as suitable initiators, a strikingly low proportion of participants were willing to be interviewed by scientific or non-governmental institutions. Again, this could be interpreted as an existing high trust in current healthcare structures but also underlines the necessity to promote scientific institutes and non-governmental institutions as reliable partners in research and patient support. Interestingly, even though participants of this study appeared to have sufficient digital media literacy, most preferred to be interviewed by letter or phone call in future studies. Stratification for digital media usage revealed that email was preferred mainly by those who used digital media for more than 1 h per day. This finding strengthened the assumption that also in stroke, subpopulations exist that can be better interviewed when using digital media. Conversely, this also means that a significant proportion of stroke survivors who do not use digital services cannot be reached by an exclusively electronic survey (ePROMs). Moreover, the overall preferred way of communication by letter could have been biased by the survey itself being carried out with a paper-based questionnaire, and answers could have been given mainly by those confirming with this way of PROMs collection.

Intending to achieve sufficient adherence in future studies focusing on PROMs, it would be helpful to consider the preferences and to overcome barriers described in this study. Personalized administration processes in PROMs research might facilitate systematic surveys while considering individual preferences. Certain perspectives on ischemic stroke seemed to be associated with social or clinical factors. Addressing these individual preferences might play a pivotal role in conducting successful surveys, especially when trying to increase response rates in stroke survivors [13]. Furthermore, considering individual preferences might also improve the validity and reliability of studies focusing on PROMs, as patients’ unmet needs and deficits in the stroke landscape become more visible when feedback is available from most patients. Although the findings of this study are not necessarily applicable to other assessments along patients’ journeys, such as patient-reported experience measures (PREMs) [28], some aspects might also be relevant for these approaches and might thus help to achieve a broader perspective regarding individual perceptions and outcomes [29].

Remarkably, in the present study, participants expressed the wish to be also informed about the results of studies dealing with HRQoL of individuals affected by stroke. This information should be of particular interest to professionals in stroke care and research as it indicates an existing need for patient participation. According to the answers given, patients should be involved in planning PROMs-related research, e.g., by developing HRQoL questionnaires, and should be informed about the study results. The findings of the present study support the earlier-mentioned perspective that a more patient-oriented approach could help to increase validity and reliability in stroke research [30].

This study has some limitations. Caused by the study design, there might be a selection bias regarding the perspectives expressed by participants. Even though more than 50% of persons who were contacted after stroke replied, it is likely that these participants showed a rather favorable attitude towards PROMs surveys, because they decided to participate in the study. Hence, no conclusion can be drawn about non-responders as this study considered data, e.g., demographic details, only from patients who replied to the survey. At time of hospital discharge, the patients included in this study exhibited no or only minor symptoms due to stroke as indicated by a low mRS and NIHSS, allowing an interpretation of findings only to this subgroup. This selection is naturally linked to the performed paper-based survey. Therefore, future initiatives should address the challenge of getting feedback from patients who cannot respond in a written form due to stroke-related symptoms. They might also include relatives or caregivers, as it would probably result in broader feedback as well as a higher mRS and NIHSS of affected persons than depicted in this study. Additionally, this would probably allow to take in account the variation of the NIHSS that is typically associated with the location of stroke, i.e., involving the anterior vs. posterior circulation.

## Conclusion

Although the findings of this study need to be confirmed in a larger cohort of stroke patients, including those more severely affected by the ischemic event, to the authors’ knowledge, this is the first approach investigating the patient’s perspective regarding the assessment of PROMs. Thereby, the obtained insights concerning the preferred way, time interval, duration, and initiation site of respective surveys add valuable information to the question of how PROMs might be best conducted to achieve high response rates, which are essential for detecting individual needs and deficits in stroke care. As a further insight, patients should be involved more frequently in planning stroke research and results should consequently be disseminated within this collective.

### Supplementary Information

**Additional file 1. Supplementary Material:** English version of questionnaire and summary of responses.

## Data Availability

The datasets generated and/or analyzed during the current study are not publicly available to preserve the anonymity of study participants but anonymized datasets are available from the corresponding author on reasonable request.

## Abbreviations

PROMs: Patient-reported outcome measures
HRQoL: Health-related quality of life
PREMs: Patient-reported experience measures
VAS: Visual analogue scale
NIHSS: National Institutes of Health Stroke Scale
GP: General physician
mRS: Modified Rankin Scale

## References

[1] Feigin VL, Brainin M, Norrving B (2022). World Stroke Organization (WSO): global Stroke fact sheet 2022. Int J Stroke.

[2] Kelly DM, Feld J, Rothwell PM (2022). Admission rates, time trends, risk factors, and outcomes of ischemic and hemorrhagic stroke from german nationwide data. Neurology.

[3] Wafa HA, Wolfe CDA, Emmett E (2020). Burden of stroke in Europe: thirty-year projections of incidence, prevalence, deaths, and disability-adjusted life years. Stroke.

[4] Bergersen H, Frøslie KF, Stibrant Sunnerhagen K, Schanke A-K (2010). Anxiety, depression, and psychological well-being 2 to 5 years poststroke. J Stroke Cerebrovasc Dis.

[5] Hartman-Maeir A, Soroker N, Ring H (2007). Activities, participation and satisfaction one-year post stroke. Disabil Rehabil.

[6] van de Port IGL, Kwakkel G, van Wijk I, Lindeman E (2006). Susceptibility to deterioration of mobility long-term after stroke: a prospective cohort study. Stroke.

[7] van Mierlo ML, van Heugten CM, Post MWM (2016). Quality of life during the first two years post stroke: the Restore4Stroke cohort study. Cerebrovasc Dis.

[8] Norrving B, Barrick J, Davalos A (2018). Action plan for stroke in Europe 2018–2030. Eur Stroke J.

[9] Katzan IL, Thompson NR, Uchino K, Lapin B (2018). The most affected health domains after ischemic stroke. Neurology.

[10] Gallacher KI, Quinn T, Kidd L (2019). Systematic review of patient-reported measures of treatment burden in Stroke. BMJ Open.

[11] Cramer SC (2020). Issues important to the design of stroke recovery trials. Lancet Neurol.

[12] Cramer SC, Wolf SL, Saver JL (2021). The utility of domain-specific end points in acute stroke trials. Stroke.

[13] Glimmerveen A, Holewijn S, Vermeer S (2023). Association between clinician reported outcome and patient reported outcome measures one year after stroke. J Stroke Cerebrovasc Dis.

[14] Salinas J, Sprinkhuizen SM, Ackerson T (2016). An international standard set of patient-centered outcome measures after stroke. Stroke.

[15] Arwert HJ, Oosterveer DM, Schoones JW (2022). Use of patient-reported outcomes measurement information system measures in clinical research in patients with stroke: a systematic literature review. Arch Rehabil Res Clin Transl.

[16] Wong MCS, Lao XQ, Ho K-F (2017). Incidence and mortality of Lung cancer: global trends and association with socioeconomic status. Sci Rep.

[17] Reeves MJ, Bushnell CD, Howard G (2008). Sex differences in stroke: epidemiology, clinical presentation, medical care, and outcomes. Lancet Neurol.

[18] Fletcher J, Swift A, Hewison M, C Cooper S (2021). Patient and public involvement in research design and oversight. Nurse Res.

[19] Hall P, Kroll T, Hickey J (2021). Patient and public involvement in stroke research: a scoping review protocol. HRB Open Res.

[20] Harrison M, Palmer R (2015). Exploring patient and public involvement in stroke research: a qualitative study. Disabil Rehabil.

[21] Vandenbroucke JP, von Elm E, Altman DG (2007). Strengthening the reporting of observational studies in epidemiology (STROBE): explanation and elaboration. PLoS Med.

[22] Lyden PD (2020). Measuring outcome after stroke: more lessons learned again. Stroke.

[23] van Swieten JC, Koudstaal PJ, Visser MC (1988). Interobserver agreement for the assessment of handicap in Stroke patients. Stroke.

[24] Rabin R, de Charro F (2001). EQ-5D: a measure of health status from the EuroQol Group. Ann Med.

[25] Ludwig K, Graf Von Der Schulenburg J-M, Greiner W (2018). German value set for the EQ-5D-5L. PharmacoEconomics.

[26] R Core Team (2022). R: a language and environment for statistical computing.

[27] RStudio T, RStudio (2022) PBC, Boston, MA

[28] Cornelis C, den Hartog SJ, Bastemeijer CM (2021). Patient-reported experience measures in stroke care: a systematic review. Stroke.

[29] Porter ME (2010). What is value in health care?. N Engl J Med.

[30] Reeves M, Lisabeth L, Williams L (2018). Patient-reported outcome measures (PROMs) for acute stroke: rationale, methods and future directions. Stroke.

