# Supplementary material for: Stroke survivors’ preferences on assessing patient-reported outcome measures
Source: J Patient Rep Outcomes. 2023 Nov 30;7:124. doi: 10.1186/s41687-023-00660-1 (PMC10689585; doi:10.1186/s41687-023-00660-1)
Supplement: Supplementary file 1 — Additional file 1. Supplementary Material: English version of questionnaire and summary of responses. [file 41687_2023_660_MOESM1_ESM.docx]

**Supplemental Material**

**Title:** Stroke survivors’ preferences on assessing patient-reported outcome measures

1. **Translation of questions regarding the assessment of PROMs**

**The number of positive responses and their proportion of all responses given to the respective question are shown in blue font.**

*Do you think a survey about the quality of life after stroke is useful, if the results can be used to improve care for those affected by stroke in the future?*

Yes **(n=66; 83.54%)**

No **(n=2; 2.53%)**

No preference **(n=11; 13.92%)**

*Do you think a survey about the quality of life after stroke is useful, if it results in a direct improvement for your personal care?*

Yes **(n=53; 71.62%)**

No **(n=4; 5.40%)**

No preference **(n=17; 22.97%)**

*What form of response options would you prefer in such a survey?*

Open-ended questions, meaning no predetermined answer choices **(n=18; 23.08%)**

Closed multiple choice question **(n=55; 70.51%)**

No preference **(n=5; 6.41%)**

*Would you participate in a survey about your quality of life after stroke if you did not receive any money or other compensation for doing so?*

Yes **(n=68; 86.08%)**

No **(n=6; 7.59%)**

No preference **(n=5; 6.33%)**

*Would you participate in a survey about your quality of life after stroke if you received money or other compensation for it?*

Yes **(n=41; 53.95%)**

No **(n=18; 23.68%)**

No preference **(n=17; 22.37%)**

*At what time point do you think a survey about quality of life after stroke would be useful? (Multiple answers possible)*

Never **(n=2; 2.50%)**

During the stay in the hospital shortly after the stroke **(n=21; 26.25%)**

During rehabilitation **(n=22; 27.5%)**

After completion of rehabilitation **(n=38; 47.5%)**

Half a year after the stroke **(n=15; 18.75%)**

One year after the stroke **(n=30; 37.5%)**

Annually after the stroke **(n=39; 48.75%)**

No preference **n=2; 2.5%)**

*At what frequency should a post-stroke quality of life survey be conducted?*

Once **(n=13; 17.11%)**

In regular intervals **(n=55; 72.37%)**

No preference **(n=8; 10.53%)**

*How much time would you take for a survey about your quality of life after stroke in the future?*

None **(n=2; 2.53%)**

Less than 2 minutes **(n=0)**

Less than 5 minutes **(n=1; 1.27%)**

Less than 15 minutes **(n=17; 21.52%)**

15 to 30 minutes **(n=41; 51.90%)**

More than 30 minutes **(n=13; 16.46%)**

No preference **(n=5; 6.33%)**

*How would you like to be interviewed in the future? (Multiple answers possible)*

In written form **(n=69; 86.25%)**

via letter **(n=58; 72.50%)**

digital, mobile app for cell phone **(n=4; 5.00%)**

digital, email **(n=17; 21.25%)**

digital, website **(n=1; 1.25%)**

digital, SMS / messenger service (e.g., WhatsApp) **(n=1; 2.5%)**

In personal conversation **(n=56; 70.00%)**

by telephone **(n=20; 25.00%)**

by video telephony **(n=2; 2.50%)**

In person at my home **(n=5; 6.25%)**

At my general practitioner’s office **(n=18; 22.50%)**

At the clinic that treated me for the stroke **(n=28; 35.00%)**

No preference **(n=2; 2.50%)**

*Who should conduct a survey on quality of life after stroke? (Multiple answers possible)*

Family doctor **(n=32; 40.51%)**

Specialist physician **(n=22; 27.85%)**

Stroke nurse / pilot **(n=28; 35.44%)**

The hospital that treated me for the stroke **(n=43; 54.43%)**

Non-profit organization in the health care system **(n=4; 5.06%)**

Scientific organization (e.g., university) **(n=10; 12.66%)**

Commercial provider (e.g., opinion research company) **(n=0)**

No preference **(n=3; 3.80%)**

*Should another person you know be asked for opinions about your personal quality of life after stroke? (Multiple answers possible)*

Yes **(n=47; 59.49%)**

Who

Family/relatives **(n=41; 51.90%)**

Family doctor/physician **(n=15; 18.99%)**

Nursing service **(n=1; 1.27%)**

No **(n=28; 35.44%)**

No preference **(n=5; 6.33%)**

*Should relatives and caregivers also be asked for their respective quality of life?*

Yes **(n=26; 35.14%)**

No **(n=35; 47.30%)**

No preference **(n=13; 17.57%)**

*In your opinion, which of the following points could make it more difficult to ask stroke patients about their quality of life? (Multiple answers possible)*

Health of the persons affected by stroke symptoms **(n=39; 48.75%)**

Missing motivation/interest of the affected persons **(n=23; 28.75.%)**

Too time-consuming **(n=10; 12.50%)**

Data protection / privacy **(n=11; 13.75%)**

No preference **(n=17; 21.25%)**

*Should participants be informed about the results of surveys on quality of life after stroke?*

Yes **(n=57; 74.03%)**

No **(n=7; 9.09%)**

No preference **(n=13; 16.88%)**

*Should people affected by stroke be involved in the design of future surveys (e.g., in the development of questions)?*

Yes **(n=30; 38.96%)**

No **(n=23; 29.87%)**

No preference **(n=24; 31.17%)**
